# Quality of Patient Information on Allergic Rhinitis in Children on the Internet

**DOI:** 10.3390/children12111432

**Published:** 2025-10-23

**Authors:** Nikolaos Kitsos, Aspasia Michoula, Ioanna Grivea

**Affiliations:** Department of Pediatrics, Faculty of Medicine, University of Thessaly, 41500 Larissa, Greece; amichoula@uth.gr (A.M.); iogrivea@uth.gr (I.G.)

**Keywords:** patient information, internet, allergic rhinitis, children, EQIP tool, health literacy, quality of life, digital health

## Abstract

**Highlights:**

**What are the main findings?**

• The majority (82%) of websites providing information on allergic rhinitis in children are of low quality, with a median EQIP score of 16.5/34.
• Academic and industry-affiliated websites scored significantly higher than practitioner-developed sites, many of which lacked essential content and transparency, including quality-of-life information.

**What is the implication of the main finding?**

• Families seeking online information about pediatric allergic rhinitis are often exposed to incomplete or unreliable content, which may affect disease understanding, diagnosis, and management.
• There is an urgent need for standardized, evidence-based, and patient-centered online resources, ideally developed collaboratively by academic institutions, healthcare professionals, and patient organizations.

**Abstract:**

**Background**: The vast majority of patients, considering information for allergic conditions, use the Internet as a source of health information. The aim of our study is to assess the quality of patient information on allergic rhinitis available on the internet. **Methods**: Three hundred Websites, found through the most recognizable search engines, were evaluated using the modified Ensuring Quality Information for Patients (EQIP) instrument. **Results**: Eighty-five websites were assessed after the exclusion of duplicates and Websites in languages other than English. Websites that scored higher than 21 (over the 75th percentile) were categorized as high-score sites. Websites that were developed by health professionals tended to have a higher score. The EQIP score of the websites ranged between 5 and 26 out of the total of 34 points, with a median value of 16.5 points. **Conclusions**: The quality of patient information on allergic rhinitis on the Internet is inferior, and the existing Websites present insufficient information.

## 1. Introduction

Over the past two decades, the Internet has become one of the most frequently used sources of health information by patients and caregivers [1]. Surveys indicate that more than two-thirds of families consult online resources before or after medical visits, often using them to complement professional advice [2]. This increasing reliance on digital platforms has transformed the physician–patient relationship, creating both opportunities and challenges [3]. Reliable online health information can empower families, enhance disease awareness, support shared decision-making, and improve adherence to treatment [4,5,6,7]. However, the unregulated nature of the Internet allows the proliferation of inaccurate, biased, or commercially motivated content, which may mislead patients and even delay appropriate care [8,9,10,11].

Allergic rhinitis (AR) is among the most prevalent chronic conditions in children, affecting approximately 13% of the global pediatric population [12]. Allergic rhinitis most commonly involves IgE-mediated inflammation; however, non-IgE-mediated immunological pathways may also contribute to its clinical expression. It is triggered by environmental allergens such as pollen, house dust mites, or animal dander [11]. Clinical manifestations typically include nasal congestion, rhinorrhea, sneezing, and itching, symptoms that may substantially impair quality of life, sleep quality, and school performance [13,14]. In younger children, diagnosis is often complicated by overlapping features with viral infections and limited ability to describe symptoms [15,16,17]. As a result, caregivers frequently turn to the Internet for guidance regarding disease recognition, management strategies, and prognosis. Recent epidemiological reports (2021–2024) indicate that allergic rhinitis remains one of the most prevalent chronic inflammatory disorders in childhood, with increasing incidence linked to urbanization and environmental exposure [18,19]. Updated ARIA and EAACI guidelines emphasize early recognition, digital literacy in allergy education, and patient-family engagement in decision-making. Furthermore, emerging studies highlight that caregivers increasingly rely on digital health platforms rather than clinical consultations for initial guidance, reinforcing the need for accurate and accessible online pediatric allergy resources.

Although online health information is widely available, its quality is highly variable. Tools such as the Ensuring Quality Information for Patients (EQIP) score have been developed to evaluate patient-directed resources in terms of accuracy, clarity, structure, and transparency [20,21]. These instruments, including the EQIP checklist, provide a standardized manual evaluation approach, where reviewers apply predefined criteria to assess the quality of patient information materials rather than relying on automated online analysis. The EQIP tool is a validated checklist-based evaluation instrument developed to assess the clarity, completeness, and transparency of patient-directed health information materials. It is not an online automated tool, but a structured evaluation framework applied manually by reviewers. It has previously been applied in evaluations of online resources in various medical conditions, including asthma, food allergy, and chronic disease education. Given the anticipated global rise in allergic diseases, ensuring that families have access to accurate and comprehensible information on pediatric AR is of increasing importance.

To date, no systematic evaluation has focused specifically on the quality of Internet-based patient information on allergic rhinitis in children. The aim of this study was therefore to conduct a systematic review of websites providing information on pediatric AR, using the modified EQIP tool to assess their content, structure, and reliability.

## 2. Methods

### 2.1. Protocol and Reporting

This systematic review was designed and reported in accordance with the Preferred Reporting Items for Systematic Reviews and Meta-Analyses (PRISMA 2020) statement. The review protocol was registered with the Open Science Framework (OSF) and is available at https://doi.org/10.17605/OSF.IO/CSWN, accessed on 26 September 2025.

### 2.2. Eligibility Criteria

We aimed to evaluate the quality of online patient-directed information regarding allergic rhinitis in children. Websites were considered eligible if they met the following criteria: (i) they contained written material specifically targeted at patients or caregivers of children with allergic rhinitis; (ii) the information was freely accessible without subscription or password protection; and (iii) the content was in English. Websites were excluded if they were duplicates, if “allergic rhinitis” was only mentioned in an unrelated context, if the primary material was non-textual (e.g., stand-alone videos without accompanying text), or if the site was published in a language other than English.

### 2.3. Information Sources

To ensure comprehensive coverage of patient-accessible online content, searches were conducted between 1 December 2023, and 31 January 2024 in the three most widely used search engines globally—Google, Bing, and Yahoo. Together, these engines account for more than 99% of search traffic in Greece and approximately 96% worldwide, making them highly representative of the platforms most frequently used by patients and caregivers seeking health information [22,23]. Academic databases such as PubMed and SpringerLink were not included in this search strategy because our objective was to reflect real-world patient and caregiver behavior, which predominantly relies on general search engines rather than academic platforms.

### 2.4. Search Strategy

The search string was standardized across platforms and consisted of the phrase: “allergic rhinitis in children.” For each engine, the first 100 consecutive results were collected, generating a total of 300 links. This strategy reflects user behavior, as previous studies have demonstrated that the majority of internet users rarely navigate beyond the first few pages of search results when retrieving health information [22,23,24,25].

### 2.5. Selection Process

All 300 websites were exported into a database for screening. A single reviewer (NK) independently assessed each entry for eligibility in two stages. First, duplicate entries were removed. Second, the remaining links were screened for relevance, accessibility, language, and format. Following this stepwise process, 85 websites fulfilled all inclusion criteria and were retained for detailed analysis.

**Figure 1:**
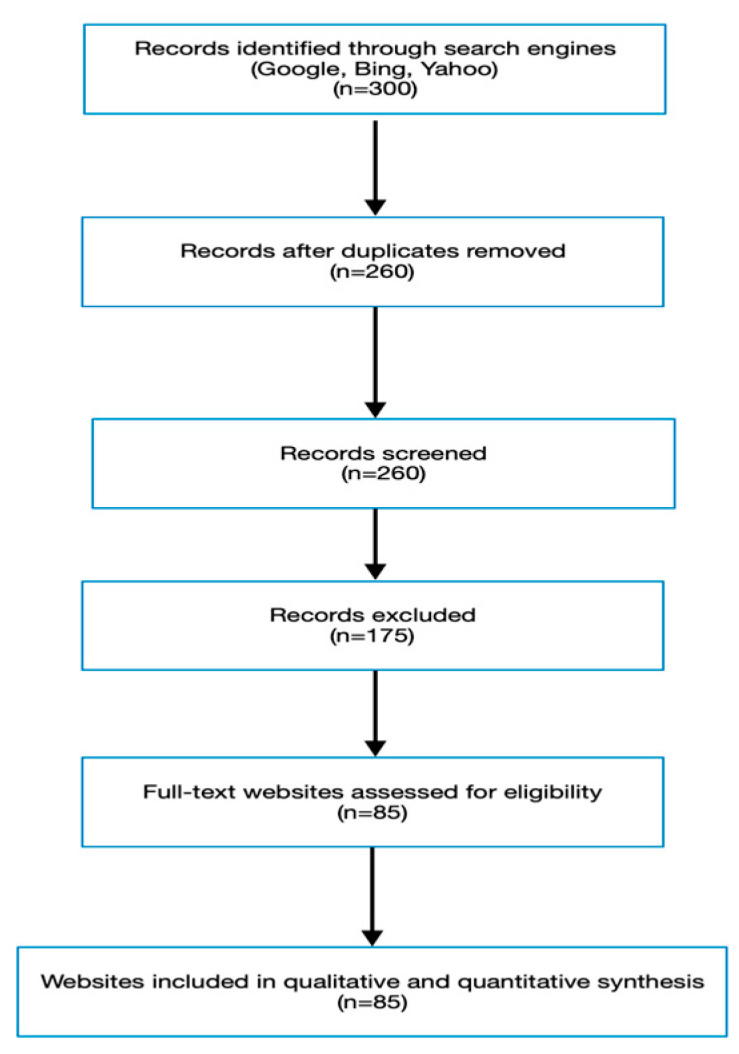
Flow diagram of website collection according to PRISMA.

The screening and selection process is summarized in the PRISMA flow diagram (Figure 1).

### 2.6. Data Collection and Website Classification

To ensure methodological consistency, data were extracted using a piloted standardized form. The reviewer first tested the form on 10 randomly selected websites and refined it before applying it to the entire dataset. Eligible websites were then categorized into ten predefined groups, consistent with prior research [25]: academic/educational institutions, encyclopedias, health departments, hospitals, industry-related sources, news services, patient advocacy groups, private practitioners, professional societies, and general web portals. This classification permitted subgroup comparisons of quality scores across different types of content providers.

In addition, potential bias was assessed qualitatively by examining website ownership, sponsorship, and transparency of authorship.

Only the main landing page of each website retrieved by the search engines was evaluated. Secondary hyperlinks or external links embedded within the website were not followed, in order to reflect typical user navigation behavior.

### 2.7. Data Items and Outcomes

The following data were collected from each eligible website: type of provider, ownership or sponsorship, presence of authorship and references, scope of medical information, and inclusion of health-related quality of life (HRQoL) considerations. The primary outcome was information quality, measured using a validated scoring system. Secondary outcomes included the distribution of websites across provider categories and the frequency with which HRQoL aspects were discussed.

### 2.8. Quality Assessment

The quality of information was assessed manually using the modified Ensuring Quality Information for Patients (EQIP) checklist instrument, with predefined criteria applied by the reviewer rather than through automated online analysis [26,27]. The EQIP tool evaluates patient materials across three domains: content (accuracy, completeness, relevance), identification (authorship, date of publication, references, conflicts of interest), and structure (clarity, readability, illustrations, and navigability) (Table 1).

### 2.9. Assessment of Bias

Risk of bias at the source level was qualitatively evaluated by examining the ownership and sponsorship of the websites as well as transparency in authorship and citation practices.

### 2.10. Statistical Analysis

Extracted data were entered into Excel and subsequently imported into SPSS version 21 (IBM Corp., Armonk, NY, USA) for statistical analysis. Descriptive statistics, including medians, interquartile ranges (IQR), and percentages, were calculated for EQIP scores. Differences between categorical variables were tested using chi-square or Fisher’s exact test, while continuous variables were compared using one-way ANOVA. A *p*-value of <0.05 was considered statistically significant. Continuous EQIP scores are reported as Mean ± SD and Median (IQR) to allow both parametric and distribution-based interpretation, while categorical variables are expressed as counts and percentages.

## 3. Results

### 3.1. Study Selection

The initial search identified 300 websites (100 each from Google, Bing, and Yahoo). After removal of duplicates, non-English entries, irrelevant content, and non-textual sources, 85 websites met all inclusion criteria and were included in the final review. The screening and selection process is summarized in the PRISMA flow diagram (Figure 1).

### 3.2. Characteristics of Included Websites

The distribution of websites across the ten predefined categories is shown in Table 2. Almost half (43.5%) were developed by private medical practitioners, making this the most common category. General portals accounted for 18.8%, while academic or educational institutions contributed 14.1%. The healthcare industry represented 7.1%, and patient advocacy groups 5.9%. Smaller proportions originated from media outlets (4.7%) and hospitals (2.4%), with even fewer from encyclopedias, news services, or professional societies. This distribution underscores that the majority of available online resources originate from individual practitioners and commercial portals rather than academic or institutional bodies.

### 3.3. Evaluation of Website Quality

The median modified EQIP score across all websites was 16.5 (IQR: 13–19), with observed scores ranging from 5 to 26 out of a possible 34. Using the 75th percentile threshold (≥21 points), only 18% of websites were classified as high quality, whereas the remaining 82% fell into the low-quality category. None of the evaluated resources achieved the maximum score. The highest-scoring website (26/34) was affiliated with an open-access academic journal, while the lowest-scoring (5/34) lacked identifiable authorship, references, and structural clarity (Table 3).

Marked variability was observed between website groups. Academic centers and industry-sponsored pages generally achieved higher scores than practitioner-developed websites. Notably, 83.5% of practitioner websites were rated low-quality (Table 3). Importantly, websites that appeared earlier in search engine results were not necessarily those with higher EQIP scores. Several high-ranking sites were evaluated as low quality, indicating that search visibility does not equate to information reliability.

### 3.4. Reporting of Health-Related Quality of Life (HRQoL)

Discussion of HRQoL was inconsistent across websites. Nearly half (49.5%) of practitioner-authored sites failed to mention quality-of-life considerations, whereas academic centers and patient advocacy groups more frequently acknowledged the burden of allergic rhinitis on children’s daily functioning, sleep, school performance, and emotional well-being [28,29,30,31,32]. Although symptoms such as nasal congestion, rhinorrhea, sneezing, and itching were universally described, only a minority of websites explicitly addressed their differential impact on children’s quality of life [33,34,35,36,37,38].

## 4. Discussion

The present study systematically evaluated the quality of online information about allergic rhinitis (AR) in children using the modified Ensuring Quality Information for Patients (EQIP) instrument. Our findings highlight major variability in content quality across different website categories and reveal that the majority of resources accessible to families are of low quality. Importantly, nearly half of the websites produced by private practitioners failed to mention the impact of AR on quality of life (QoL), suggesting a lack of emphasis on patient-centered outcomes. To our knowledge, this is the first systematic review to specifically assess web-based patient information regarding pediatric AR.

Our results align with earlier research investigating online health information for other chronic pediatric conditions. For example, studies on asthma, atopic dermatitis, and food allergy websites have consistently demonstrated suboptimal content quality and a predominance of non-peer-reviewed sources [39,40]. In each of these conditions, websites developed by academic centers or professional organizations tended to achieve higher quality scores compared with those authored by individual practitioners or commercial entities. The present study extends this pattern to pediatric AR, confirming that information provided by authoritative institutions remains more reliable than that generated by private actors.

Interestingly, the median EQIP score of 16.5 in our sample is comparable to values reported in systematic reviews of other allergic diseases [41,42]. However, the upper range of scores (26/34) was somewhat lower than maxima reported in evaluations of asthma websites, where a small number of resources reached near-complete compliance with evaluation tools. This suggests that online content related to AR may be particularly underdeveloped in terms of completeness and structure.

The low overall quality of websites poses important challenges for families seeking guidance. Pediatric AR is a highly prevalent condition, affecting nearly one in eight children worldwide, and parents often rely on digital resources for reassurance or decision-making. Inaccurate or incomplete online information may delay diagnosis, promote inappropriate self-treatment, or undermine adherence to evidence-based management plans.

The absence of adequate coverage of QoL issues is of particular concern. Pediatric AR has well-documented effects on school performance, sleep quality, and psychosocial well-being. Failure to highlight these outcomes may mislead caregivers into underestimating the burden of the disease. Furthermore, neglecting QoL considerations reduces opportunities for shared decision-making, which is increasingly recognized as central to family-centered pediatric care.

Our results also suggest that practitioner-developed websites—despite representing the largest proportion of online resources—frequently score poorly in terms of structure, authorship transparency, and content completeness. This discrepancy may reflect limited expertise in digital communication among individual clinicians, lack of dedicated editorial oversight, or the absence of standardized templates for patient education materials.

The modified EQIP tool proved useful for systematically capturing variability in website quality. By assessing three key domains—content, identification, and structure—it provided a balanced evaluation of both accuracy and usability. The decision to exclude the “partially yes” category enhanced reproducibility by reducing subjectivity, consistent with previous methodological recommendations. While the EQIP tool does not address all aspects of digital health literacy, such as interactivity or cultural appropriateness, its structured framework facilitated clear comparisons across website categories.

Several limitations of our study should be acknowledged. First, only English-language websites were included, meaning the quality of resources available in other languages remains unassessed. This may disproportionately affect families in non-English-speaking regions, where reliance on locally developed materials may be higher. Second, our search strategy used a single keyword (“allergic rhinitis”), which, while representative of typical patient searches, may have excluded relevant resources retrieved by alternative terms such as “hay fever in children” or “seasonal allergies.” Third, evaluation was performed by a single investigator, raising the possibility of selection or scoring bias, although the binary scoring system of the modified EQIP instrument likely minimized subjectivity. This introduces a potential subjectivity bias, and future studies should incorporate at least two independent reviewers to increase inter-rater reliability. Furthermore, we acknowledge as a methodological limitation that although academic databases such as PubMed or SpringerLink may host higher-quality patient information materials, these platforms are not typically accessed by the general public. Therefore, they were intentionally excluded to maintain a patient-centered approach focused on freely accessible, real-world information-seeking behavior.

Another limitation is that the EQIP tool, while validated, was not designed exclusively for allergic conditions. Some aspects of AR-specific patient education, such as allergen avoidance techniques, immunotherapy options, or comorbidity management, may not have been fully captured. Additionally, the Internet is a dynamic medium: search rankings evolve rapidly, and websites may be updated or removed. Our findings therefore represent a snapshot of available resources during the search period rather than a static evaluation.

Given the widespread reliance of families on digital health information, urgent steps are needed to improve the quality of online content related to pediatric AR. A multidisciplinary approach involving pediatric allergists, patient advocacy groups, communication specialists, and digital health experts is likely required. Several strategies may be considered. Firstly, standardization of web content: Development of consensus-based templates for AR educational materials, incorporating both clinical and QoL dimensions, could guide practitioners and institutions in creating reliable online resources. Additionally, accreditation or Quality Labels: Websites could display a certification tag, such as “Reviewed with the EQIP tool,” signaling to families that content has undergone quality assurance. Similar models, such as the Health On the Net (HON) code, have been shown to increase patient trust. Furthermore, integration into clinical care: pediatricians and allergists should actively direct families to high-quality online resources during consultations. Doing so not only ensures that patients access reliable information but also strengthens the doctor–patient relationship by framing the Internet as a complementary tool rather than a competing authority. Expansion of Multilingual Resources: Considering the global burden of AR, the creation of high-quality, culturally adapted, and multilingual websites should be prioritized.

Future studies should expand beyond English-language content, involve multiple evaluators to confirm reproducibility, and consider complementary tools such as the International Patient Decision Aids Standards (IPDAS) checklist to capture decision-support features. Longitudinal studies may also be valuable to assess how the quality of online AR resources evolves over time.

## 5. Conclusions

This systematic review demonstrates that the majority of online resources addressing allergic rhinitis in children are of low quality according to the modified EQIP instrument. Academic and industry-affiliated websites generally performed better than practitioner-developed sites, which frequently lacked essential content and transparency. Particularly concerning was the omission of health-related QoL information in nearly half of the evaluated websites, limiting their relevance for patient-centered care.

Improving the quality, completeness, and accessibility of digital patient information on pediatric AR is an urgent priority. Given the projected rise in allergic diseases worldwide, efforts should focus on the creation of standardized, evidence-based, and user-friendly resources that empower families while supporting shared decision-making. Collaboration among healthcare professionals, professional societies, and patient organizations will be essential to achieve this goal.

## Figures and Tables

**Table 1:** Overall results of the included Websites according to the Ensuring Quality Information for Patients instrument (EQIP instrument).

Item	Criteria	Yes (%)	No (%)	Does Not Apply (%)
Content data				
1	Initial definition of which subjects will be covered	60 (70.5)	25 (29.5)	0 (0)
2	Coverage of the previously defined subjects	63 (74.1)	2 (2.4)	20 (23.5)
3	Description of the medical problem	61 (71.8)	24 (28.2)	0 (0)
4	Description of treatment alternatives	66 (77.6)	19 (22.4)	0 (0)
5	Description of the qualitative benefits to the recipient	37 (43.5)	48 (56.5)	0 (0)
6	Description of the quantitative benefits to the recipient	60 (70.5)	25 (29.5)	0 (0)
7	Description of the qualitative risks and side effects	44 (51.8)	41 (48.2)	0 (0)
8	Description of the quantitative risks and side effects	27 (31.8)	58 (68.2)	0 (0)
9	Addressing quality of life issues	25 (29.4)	60 (70.6)	0 (0)
10	Description of how complications are handled	23 (27)	62 (73)	0 (0)
11	Description of the precautions that the patient may take	14 (16.5)	71 (83.5)	0 (0)
12	Mention of alert signs that the patient may detect	19 (22.4)	66 (77.6)	0 (0)
13	Addressing medical intervention costs and insurance issues	24 (28.2)	61 (71.8)	0 (0)
14	Specific contact details for hospital services	58 (68.2)	27 (31.8)	0 (0)
15	Specific details of other sources of reliable information/support	32 (37.6)	53 (62.4)	0 (0)
16	Coverage of all relevant issues for the topic	48 (56.5)	37 (43.5)	0 (0)
Identification data				
17	Date of issue or revision	27 (31.8)	58 (68.2)	0 (0)
18	Logo of the issuing body	59 (69.4)	26 (30.6)	0 (0)
19	Names of the persons or entities that produced the document	14 (16.5)	71 (83.5)	0 (0)
20	Names of the persons or entities that financed the document	2 (2.4)	83 (97.6)	0 (0)
21	Short bibliography of the evidence-based data used in the document	12 (14.1)	73 (85.9)	0 (0)
22	Statement about whether and how patients were involved/consulted in the document’s production	1 (1.2)	84 (98.8)	0 (0)
Structure data				
23	Use of everyday language and explanation of complex words or jargon	82 (96.5)	3 (3.5)	
24	Use of generic names for all medications or products	3 (3.5)	1 (1.2)	81 (95.3)
25	Use of short sentences (>15 words on average)	85 (100)	0 (0)	0 (0)
26	Personal address to the reader	54 (63.5)	31 (36.5)	0 (0)
27	Respectful tone	85 (100)	0 (0)	0 (0)
28	Clear information (no ambiguities or contradictions)	73 (85.9)	12 (14.1)	0 (0)
29	Balanced information on risks and benefits	32 (37.6)	53 (62.4)	0 (0)
30	Presentation of information in a logical order	72 (84.7)	13 (15.3)	0 (0)
31	Satisfactory design and layout (excluding figures or graphs)	77 (90.6)	8 (9.4)	0 (0)
32	Clear and relevant figures or graphs	16 (18.8)	0 (0)	69 (81.2)
33	Inclusion of a named space for the reader’s note or questions	47 (55.3)	38 (44.7)	0 (0)
34	Inclusion of a printed consent form contrary to recommendation	0 (0)	2 (2.4)	83 (97.6)
Summary (n = 85): 16.82 ± 4.14 (Mean ± SD) − Median (IQR) 16.5 (13–19); Min = 5, Max = 26				

**Table 2:** Number of Websites provided by different developers.

Site’s Category	Frequency
Encyclopedias	1
Media	4
Academic	12
Practitioners	37
Hospital	2
Web portal	16
Patient groups	5
The industry	6
News services	1
Professional societies	1
Total	85

**Table 3:** Websites >95th percentile according to the EQIP instrument.

Ranking	Website	Site’s Category	Score
1	https://www.ncbi.nlm.nih.gov/pmc/articles/PMC8974858/ (accessed on 5 December 2023)	Academic	26
2	https://www.childrenshospital.org/conditions/allergic-rhinitis (accessed on 3 December 2023)	Hospital	25
3	https://www.hopkinsmedicine.org/health/conditions-and-diseases/allergic-rhinitis-in-children (accessed on 14 January 2024)	Practitioners	24
3	https://www.ncbi.nlm.nih.gov/pmc/articles/PMC10528841/ (accessed on 28 December 2023)	Academic	24

## Data Availability

The data supporting this study are available from the corresponding author upon reasonable request. The review protocol is openly available at the Open Science Framework (OSF): https://doi.org/10.17605/OSF.IO/CSWN, accessed on 26 September 2025.
